# Women's perceptions about mobile health solutions for selection and use of family planning methods in Karachi: a feasibility study

**DOI:** 10.1186/s12905-022-02086-1

**Published:** 2022-12-02

**Authors:** Farina Gul Abrejo, Romaina Iqbal, Sarah Saleem

**Affiliations:** Aga Khan University, Karachi, Pakistan

**Keywords:** mHealth, Mobile health, Family planning, Low socio-economic areas, The feasibility of family planning, Acceptability of family planning, Low and middle-income countries

## Abstract

**Background:**

The qualitative study explored the feasibility and acceptability of potential mHealth intervention for women living in low socio-economic areas to increase the uptake of family planning. The study also examined providers' perceptions' potential benefits of mHealth intervention.

**Methods:**

The qualitative exploratory study recorded the perception of 23 women and conducted seven in-depth interviews with the providers of family planning services. These interviews assessed women's attitudes through; personal experience based on the usage of smartphones and family planning, acceptability for personal benefits, features of mobile applications and the convenient language, and self-efficacy for identifying the potential impact of mHealth intervention to increase women's empowerment for family planning usage.

**Results:**

Three predetermined themes were used to record women’s perceptions. Women's personal experience identified that women in low-socioeconomic areas use mobile phones frequently and also use them for gaining information related to health. Few women have experience using mobile phones to get information about sexual and reproductive health. Women considered; poor counselling and high transport costs to the facilities as significant barriers to getting family planning services. Perceived acceptability discussed the potential features of the mHealth app. Women considered that including short videos instead of written material would help them to understand its usage and complete information about family planning methods, including; dosage, expected side effects, and potential benefits suggested to include in the app. Women perceived that the mHealth intervention would save their transport costs to the facility and fill the information gap about family planning methods.

In comparison, providers considered it would save time in counselling and motivating women at the facility. One of the significant factors discussed was self-efficacy in the form of women's empowerment in deciding on family planning. Women discussed that the mHealth intervention would increase their self-confidence to discuss the method with their husbands.

**Conclusion:**

There is a high potential for mHealth interventions for family planning in Pakistan. The usage of mobile phones can increase women's acceptability and accessibility for family planning uptake in the country.

## Introduction

"Mobile Health or mHealth" comes under the umbrella of eHealth; and defines as "the use of mobile and wireless communication technologies to improve the healthcare delivery, outcomes and research" [1, 2]. More than 60% of the population in low and middle-income countries (LMICs) use mobile phones in healthcare for diagnostic, monitoring, health reporting, surveillance etc. [1, 3]. Using mHealth to increase knowledge and uptake of modern contraceptives has been considered one of the cost-effective interventions in the world [4], especially for LMICs, where modern contraceptives change behaviour and increase uptake [5]. Several single or combined mobile phone interventions, such as text messages, voice messages, videos and applications, are used as an alternative or adjunct to face-to-face family planning services to increase the uptake of family planning in the LMICs [6]. Applications such as m4RH in Cambodia. [7], iMACC (Mobile Application for Contraceptive Choice) in Kenya[8], and in Sierra Leone for connecting the young population to family planning services are among those successful interventions. Further, a strong link exists between mHealth intervention and patient-centred care, considered the core empowerment process [2, 3]. The study by Oxford University mentioned that mobile phone usage increases women's empowerment among LMICs as it provides better information about sexual and reproductive health, which helps women to decide on their own about their sexual life [9].

According to Pakistan Demographic and Health Survey (2017–2018), only 34% of women use the method of family planning, even though the usage of the modern contraceptive method is lower (25%), and the unmet need is 17% among women of reproductive age in Pakistan. This unmet need is even higher among women living in the lowest wealth quantile, 23% in the lowest and 19% in the second-lowest wealth quantile. On the other hand, there is a forecast that the penetration of smartphones will increase to 51% in 2020, which is five times higher than in 2014 [10]. because of the increase in smartphones, using mobile health intervention in Pakistan can quickly increase family planning. A study to understand the feasibility of mHealth for improving the uptake of antenatal and postnatal services in Pakistan identified that women and providers are willing to use mHeatlh for these services because they considered it "more beneficial than face-to-face communication" [11]. mHealth has experimented in Pakistan on a small scale where the Lady Health Workers (LHWs) used the mobile application *Roshan Mustaqbil* (Bright Future). It trained and educated these workers on counselling for reproductive, maternal, newborn and child health (RMNCH) services in Sindh Province.

mHealth increases self-efficacy, an essential factor in achieving empowerment and one of the digital interventions' positive outcomes [12]. This study explored the perception of women for acceptability and feasibility of the family planning application that will provide the required information and knowledge that women need to accept the contraceptive method. The study explored the experiences and perceptions of married women of reproductive age (18–49 years) regarding the role of mHealth intervention for family planning information. The study also investigates the perception of providers (community health workers and doctors who provide family planning services) to understand the design and content of applications that could best support women in the uptake of family planning. The study also discussed the potential of mobile phone applications for increasing family planning uptake among users and providers.

## Methods

### Study design

The study used a qualitative exploratory study design. It helped to understand the perception of women regarding the feasibility and acceptability of using mobile applications to increase the uptake of family planning. This study aims to explore the general concept of using mobile applications for family planning among women living in the low socio-economic areas of Karachi.

Twenty-three semi-structured in-depth interviews were conducted with women aged 18–49. These interviews explored the current usage of family planning among women and also identified the attitude of women toward mobile phone usage. The future intention of women to use mobile phone applications to increase the uptake of family planning was also explored. Furthermore, women’s preference for mobile applications, including language, features and the information on family planning that needs to be included in the application, was also discussed during these interviews.

Seven in-depth interviews were conducted with family planning services providers (doctors, lady health visitors and nurses). The purpose of accomplishing these interviews was to record the insight of these providers into the current need for family planning among women, their perception about how women can use mobile phone applications for family planning and understand the perceived impact of mobile phone applications on family planning services.

### Study setting

The study was conducted in Azam Basti and Gadap Town of Karachi in May 2020-July 2020. Azam Basti is located in North East Karachi and has an approximate population of 120,000. There is a diverse population in Azam Basti, but most are the Christian community living in this area. At the same time, Gadap Town is in North West Karachi with around 300,000 people. Most of the people belong to the *Baloch* community in Gadap Town. These areas were selected purposively, as Aga Khan University has current projects in those areas, and the field staff has a good rapport with the population and healthcare providers. Further, the population in these areas belonging to the low socio-economic group and is highly diverse is another reason for choosing these areas. However, it was challenging to find the proportion of smartphone usage in those selected areas; the smartphone penetration is more than 18% for Pakistan, which ranked the country in the 20^th^ position for smartphone usage [15].

### Study recruitment and sampling

The field staff of projects from Aga Khan University working in the selected areas were briefed about the study's purpose and objectives. These field staff approached women of reproductive age (18–49 years) living in those areas, and gave a brief introduction of the study to those women who were married and belonged to reproductive age (18–49 years), owned a smartphone or used her husband's smartphone, and who has been used or currently using family planning. Later, these participants were approached by the study staff, who explained the study, and informed consent was obtained from them. The interviews were conducted at their houses at their convenience.

The family planning providers recruited for this study who have been providing family planning services for the last more than two years in the same area as these providers had a better understanding of local women using mobile phones and the potential of using mobile applications for family planning in the future. The providers were interviewed at their facilities at a convenient time. The purpose of provider recruitment is to understand their perception of introducing mobile applications addressing family planning information to the women they serve in the communities. As it was difficult to decide the exact number of interviews at this study's early stage, the interviews were conducted until the time of saturation [13, 14].

### The theoretical framework of the study

The Theoretical framework of this research developed after reviewing studies addressing the concepts of women's autonomy and empowerment in family planning [16] and the acceptability of family planning [17]. For the deductive approach, the theoretical framework helped to develop an interview guide. There is limited evidence of using these themes for mHealth. This framework helped to identify the factors covered under each of them. It was also essential to explore the personal experience of women regarding family planning and also smartphone usage. The study identified current/previous practices for family planning and smartphone usage (specifically for using different applications). It helped to understand what factors motivate or stop women from using family planning methods.

**Fig. 1:**
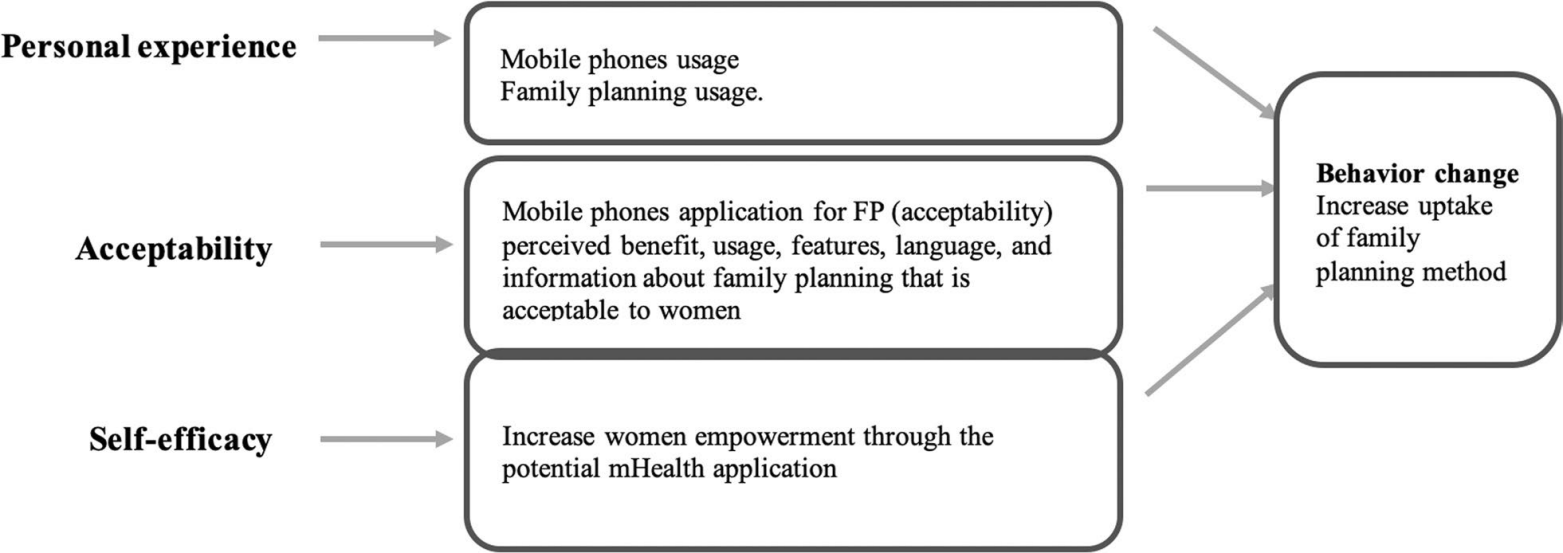
Theoretical framework of the study

On the other hand, smartphone usage, including preferred applications and features, was also explored under this domain. The second important factor was the acceptability and feasibility of mobile applications for family planning among women of reproductive age. The factors that must be considered while developing the application include language, features, etc. The third domain was self-efficacy. Empowerment, which produces a high probability of specific behaviour, results from self-efficacy [18]. In this research, empowerment is women's behaviour towards deciding on family planning. The perception of women was recorded for self-efficacy in the form of empowerment. Figure 1 describes the factors this study identified under personal experience, acceptability and self-efficacy.

### Study procedure

After getting verbal consent, the date and time were set with participants. The purpose of the study, along with the procedure explained before taking written consent. The educated participants provided written consent. A thumb impression was obtained from those who cannot read and write. The attitude of women was assessed in two areas; usage of smartphones, including timings and features of mobile phones, and current use of family planning, also explored with questions about decision-making for family planning methods, availability of family planning services, problems related to the accessibility of the services, and the information provided to the women at the time of services delivery. Acceptability and feasibility were assessed through inquiries related to potential features and preferred language of the family planning application, the kind of information women considered essential to be included in the application, and the perceived benefit was also explored through the semi-structured guideline. Finally, the self-efficacy of women for using the family planning application independently and the perceived contribution of the application towards women's empowerment was identified through questions related to the potential benefit of the application on women's life. Similarly, the in-depth interviews with providers determine the perceived attitude, feasibility and self-efficacy of women who visit the family planning facilities.

### Data analysis

The data was transcribed from the local language (Urdu) to English. NVivo 11, a qualitative data analysis software, was used for coding. As the study framework had predetermined themes. A checklist is the Consolidate Criteria for reporting qualitative research (CREQ) checklist was used to report essential aspects of research.

The rigour of the study was maintained throughout the phase of designing, conducting and reporting this research. The study used four criteria of trustworthiness; transferability, dependability and confirmability in the following manner:

1. Credibility: was maintained through “prolonged engagement” of research staff, which belong to those communities and have been working in there since last more than five years. The staff developed rapport and trust among the community members. Further, using the perspective of women living in society and recording responses from providers who are specifically providing family planning services in the same facilities helped to understand how the mobile app for family planning would be beneficial to people living in low socio-economic communities. In addition to that, “member checks” was also used to strengthen credibility, where the informal discussion with the members of the communities helped with data testing, interpreting and categorizing as well as developing conclusion with those members during the normal course of observation.
2. The external validity was focused on transferability, where the data collection method was the same for both of the sites in Karachi. Similarities and differences between those two sites were analyzed before and during the interviews. However, the study may not be generalizable in the areas with low usage of mobile phones and barriers for women using a mobile phone or accessing family planning facilities. Transferability of the research was established through “thick description”, where contextual information about the field worksite was obtained. Further, the method and timeframe of the study are described in this paper so that it would be easy for the audience to duplicate the study or generalize it in their context.
3. The dependability of this study was ensured through peer debriefing (external audits), where the expert in the field ensured the accuracy of this research. The study was shared with experts in the field to ensure self-credibility and reduce biases. Sufficient detail of the study was also incorporated to reduce problem confusion or disagreement with the data.
4. Confirmability is done through triangulation, where the data from providers and users was examined for consistency. Reflexive notes are also used to ensure the robustness of the data.

The first step was thematic content analysis, where predefined broader themes of attitude, perceived norms and personal agency were further verified, confirmed and qualified with the help of two researchers involved in 'open coding'. The words or summary of each phrase were identified, and then, information was recognized. The second step was identifying duplication and crossing out communication, which helps recognize similar categories. The list of final categories was compiled. Specific colour codes were given to each class, and information related to those particular categories was separated and summarised. The in-depth interviews increase the credibility using thick descriptions so that the study can be transferred. Reflective notes were maintained during and after the discussions. (Table 1 shows themes and types).

**Table 1:** Themes, sub-themes, categories and codes

Sub-theme	Categories	Codes
**Theme 1 personal experience**		
Mobile phone usage	Features	SMS
		Videos
		Internet searching
		Calls
		Voice messages
	Applications	WhatsApp
		Youtube
		Tiktok
		Facebook
	Timings	Morning
		Evening
	For health	COVID-19 information
		Home remedies for diarrhoea
		Irregular manustral cycle
		Sexual health issues
		Contact doctors through mobile phone
Family planning usage	Reason for using family planning	Space between children
		Don't want more children
		Malnourishment
		Poverty
	Reason for not using family planning	Side effects
		Want to have a son
**Theme 2: Acceptability of mobile application**		
Perceived benefit for women	Provide information and education	Family planning method
		Side effects and how to manage them
		Address misconceptions
	Cost-effectiveness	Travelling cost
		Doctor's fee
		Fee for side effect management
Features for mobile application	Language	Local language (Urdu)
	Features	Videos
		Pictures
		Voice recordings
Information about family planning	Family planning method	Usage
		Side effects
		Advantages
		Information about misconceptions related to the method
	Other information	Infertility
		Information regarding family planning facilities
Perceived benefits for providers		Save the time of counselling
		Women will have all the information on side effects
		Women already know the potential benefit
		With the given time, provide can serve more women
**Theme 3: self-efficacy**		
Increase empowerment		Women can make the decision on their own for family planning method
		It will help to reduce dependency on community workers and providers
		Increase self-confidence

## Results

There were 30 in-depth interviews conducted, among which 23 were married women of reproductive age living in the selected sites, and seven were family planning providers, including; LHVs (n = 4), doctors (n = 1) and Family Welfare Workers (n = 2). (Table 2: Characteristics of respondents). Most respondents own a smartphone (n = 18), and the rest use it. Most respondents own a smartphone (n = 18), and the rest use their husband's mobile phones.

**Table 2:** Respondents’ characteristics

Characteristics	Women of reproductive age	Family planning providers
**Age, years**		
18–27	5	1
28–37	11	4
38–47	7	2
**Education**		
No schooling	5	–
Grade 1–5	3	–
Grade 6–10	7	–
Intermediate	5	4
Bachelors	3	3
**Working experience**		
1–10 years	-	3
11–20 years	-	4
**Number of children**		
No children	-	2
1–3 Children	10	3
4–6 Children	13	2

Three predetermined themes helped to explore the perception of women of mobile applications, (I) personal experience on using mobile phones and currently or previously used family planning methods, (II) acceptability is the perception of respondents about the features and information that consider essential and acceptable to application and (III) self-efficacy which focused on the empowerment that women expect to achieve by using the mHealth intervention.

### Personal experience

The semi-structured guide inquired about mobile phone usage and experience related to the family planning method with women. The following sub-themes and categories emerged from the data.

### Mobile phone usage

Mobile phone usage was higher among respondents who own smartphones (5–6 h) than those who use their husbands' phones (1–2 h). The timings of using the mobile phone were also different among these respondents; owners use it in the morning and the evening, while women who use the mobile phone of their husbands use it in the evening only. All women were well aware of mobile applications and their usage; almost all use WhatsApp (a messenger application). The primary reason for using this app is that it's free of cost, and people can send voice messages and easily share files.

'I use WhatsApp because it is free, and many of my friends share videos and photos through this application (A woman living in GadapTown).

### Mobile phone usage for health

Most respondents mentioned that they use their phone to search for prevention against Coronavirus these days. Women also discussed using mobile phones to search for home remedies for diarrhoea, headache, flu or fever, and chronic diseases such as arthritis, diabetes and heart diseases.

My children mostly become sick with diarrhoea, so I sometimes search for home remedies to treat diarrhoea, and it does work (A woman from Azam Basti)

I watch YouTube videos to see prevention and treatment of diabetics and arthritis because my mother has these diseases (A woman from Azam Basti)

A few women discussed using smartphones to get information about issues related to sexual health, such as irregular menstrual cycles and heavy bleeding during menstrual. The primary reason for searching for these topics is their limited access to the hospital, which is far from their home, and they cannot visit these facilities alone. However, the usage of mobile phones for acquiring family planning information was mentioned only by two respondents, as they use mobile phones for searching for contraceptive injections, their dosage and side effects.

I use google to find home remedies for sexual issues because I cannot go to the public hospital, and it is a most convenient way to get help while staying at home (A woman from Gadap Town)

### Recent experience related to family planning

Women showed a positive attitude towards family planning. The primary reason for using the family planning method was "poverty", as they cannot afford education, and food and resources are limited.

I don't want to have more children, my husband is a worker in a factory, and we don't have enough money to raise children. My husband asked me to do family planning, and that's why I am using it (A woman from Azam Basti).

The questions explored women’s autonomy in using family planning methods. Most of the respondents considered it a "mutual decision". However, a few discussed using it without telling their husband or in-laws. Still, all of the respondents highlighted that they couldn’t decide on their own, as the consensus of the husband is considered very important.

Respondents from both sites considered transport costs a significant issue for availing of family planning services. The significant barriers were; long waiting times, provider behaviour, and poor counselling (no information) at the service delivery time.

The nurse at the facility didn't tell me much about the method; when I went there to receive services, she gave me an implant without explaining anything (A woman from Gadap Town)

The in-depth interviews also discussed the source of information for family planning with women. Few women discussed LHW as a source, but many considered peers/ friends, the facility near their home, and midwives working in the communities. Respondents highlighted that the complete information, including side effects, is not provided at the facilities, which is a significant gap.

*'The providers at the facilities are not giving complete information on side effects, which they should because women who use an implant or IUCD need to go far away for removal of these methods' (A woman from Gadap Town).*

### Acceptability of potential mHealth application

This broader theme covered the perceived acceptability of a potential mHealth application for family planning, which includes the preference for language, features, and information that needs to be included as a salient feature of the application to meet the demand for family planning. However, there is no application; the idea of a mobile application was explained to both women and providers. Applications from other countries were used for providing information to the clients [19]; clients were shown to the women and providers.

#### Information

This probe explored women's and providers' perceptions of the kind of information that needs to be included in the application. Several suggestions were discussed during the interviews, and almost all the respondents insisted on having complete information on all family planning methods. The term complete information was defined as the information about every family planning method, including its dosage, the benefit of every technique, potential side effects, and ways to manage side effects at home. A few providers suggest including the social advantage of poverty reduction as a benefit of family planning to convince people living in low-socioeconomic areas to use contraception. The majority of respondents also suggested adding information about common misconceptions and information about the facilities that provide services for method removal. Most women mentioned that there was no need for information about family planning facilities, as they knew where to go for these services. Surprisingly, a few providers suggested not including information on side effects in the potential application.

Don't put information about side effects; it will discourage the woman, and she won't use the method (An LHV from Azam Basti)

### Language

The respondents of this study belong to low socio-economic areas where the literacy level is shallow. Most of the women preferred to have content in the local language (Urdu) because many women in those communities could easily read Urdu. However, a few women in Gapad Town considered including Sindhi (another local language) in addition to Urdu for the content in the application.

### Features

All respondents were well aware of the different parts of the mobile phone application. Short videos for providing family planning information about; methods, usage, advantages and side effects were the women’s most recommended features of the application. According to the respondents (both women and providers), short videos are easy to understand, especially for illiterate women who cannot read the content. A few respondents also suggested putting pictures in the app.

### Perceived benefits by women

Several benefits were discussed during the interviews. The most important use was saving transport costs, which was discussed highly by the women.

If I get all the information at home, I don't need to spend money to go to the facility which is far away; sometimes we go there just to get information only (A woman from Gadap Town)

Once I went to the clinic, the doctor told me about the IUCD; I didn't get the service because I had to think about it and needed to discuss it with my husband. This application will save my money for such trips (A woman from Azam Basti).

Another discussed benefit was filling the information gap for family planning. According to women, most of the time, LHWs working in the area don't have complete knowledge about the family planning method. Therefore, they don't respond to women's queries. This application will be helpful to LHWs as well. '*There are many things which LHWs and health workers don't know. This application will increase their knowledge as well (A woman from Gadap Town).*

*'We have a gathering of married women in our community; if this information is available through the application, we can discuss it with each other and increase knowledge about family planning methods among women of our community (A woman from Gadap Town).*

### Perceived benefits by providers

The perceived benefits were different for providers compared to women. The most important benefit was saving providers time at the health facility. Most providers were discussed as women who will use applications and come prepared to the facility for a specific method, which will save providers time to counsel women in a particular way.

If women already have all the knowledge about the family planning method including side effects and benefits, she will come to a decision about the method, and this will save my time of counselling and convincing women about using that method' (A provider from Gadap Town).

A few providers also considered that the potential mHealth application would save providers time because they will stay at the facility due to many clients for family planning.

'The client flow is high in our facility; sometimes, we have to stay longer at the facility. This application will reduce our time and make our work easy (A provider from Azam Basti)

### Self-efficacy

Is related to the perception of respondents about increasing empowerment by making the decision on their own, less dependent on community workers, and increasing self-confidence.

It was essential to explore women's understanding of empowerment. All the women unanimously considered deciding on their own about the family planning method as an indicator of empowerment. However, these women emphasized that decision-making for contraception should be “mutual" between the husband and wife, but explaining the method to their husband along with all the advantages and disadvantages was assumed to be complicated. According to women, this application will help them to decide as they will receive all the information and can communicate with their husbands confidently.

'When I started using injection, my husband and I were confused because of all the misconceptions; this application can help me understand all the potential benefits and harms of the method. I can even show it to my husband to make him understand as well' (A woman from Gadap Town).

Women also discussed the potential application for reducing their dependency on community workers.

'The LHW in my area told me that pills are not suitable for me, but when I went to the clinic, she (the doctor) gave me pills. You know these community workers don't have complete knowledge. This application can help women (like me) to be more independent and less relying on LHWs' (A woman from Gadap Town).

Overall, the perception of the respondent for self-efficacy is associated with self-confidence. According to women, the application will increase their ability and reduce their dependency on health workers to provide information about the contraceptive method, which will eventually increase their self-confidence in making decisions about the way that (according to them) is more suitable for them.

## Discussion

The overall goal of this study was to explore the personal experience, acceptability, and self-efficacy of the potential application for increasing the uptake of family planning. The first them of individual expertise for mobile phones identified that women who own mobile phones use them more than those who use their husbands’ phones. However, women use phones for getting health-related information, while the usage of phones for acquiring family planning information is low among respondents. Women discussed two significant barriers to availing of family planning services; transport costs and poor counselling. The acceptability theme recognized that complete information details, including dosage, side effects, and potential benefits, would increase the acceptability of the mobile application. The preferred language of the application is Urdu, and using videos to provide family planning information was considered appropriate. The primary benefit of women's perception was; saving travelling costs which women do only to get information about family planning methods, and increasing women's autonomy by filling the information gap. At the same time, providers considered that the application would save their time in counselling at the facility. The self-efficacy about women's empowerment identified that the application would increase women's decision-making ability and reduce their dependency on community workers.

Providing complete information regarding family planning methods is a fundamental right of the individual [20]. During this exploratory study, women expressed enthusiasm for using the mHealth application for family planning in the future. They emphasized including complete information about family planning methods in the app to increase its acceptability. A study in a similar setting discussed that 'word of mouth is the main source of information on family planning in rural areas of Pakistan [21]. Our study participants suggested that including information about side effects for every method is important. A study on the Development and testing of the contraceptive counselling app "iPlan" also considered providing information on side effects and the contraceptive failure rate as an essential feature of the mobile application [22]. Further, the providers in this study consider using information about family planning as a poverty reduction tool; the evidence also suggests that family planning intervention increases women’s status and reduces poverty [23].

One of the critical perceived challenges by women is travelling cost, which they bear most of the time getting information about family planning methods; women suggest that they will save this cost by receiving all the information at home. Literature indicates that mobile health interventions in family planning are considered the best cost-effective strategy for LMICs [26, 27]. Poor counselling by the provider is another barrier discussed during this research, and the mHealth application is considered the best solution to overcome this obstacle. Several mobile applications such as M4RH [24], D-tree [25], and iMACC [26] are introduced to provide complete and comprehensive counselling to the users through community workers. Comprehensive counselling needs providers adequate time; it is essential for user satisfaction with contraceptive methods. In this study, the provider mentioned the mHealth intervention as a time-saving strategy for them, as women already have all the information about the contraceptive methods, which will save the provider's time to counsel and convince women about the contraceptive methods. Furthermore, the evidence shows that the family planning application saves counselling time for the providers [26].

The study identified that mHealth intervention in family planning increase women's ability to decide about family planning methods. The participant of this study felt that most lady health workers don't have adequate knowledge of family planning methods; health intervention can fill this gap. In addition to that, complete information about methods, including side effects, can help women to make decisions on their own.

Literature suggests that mHealth increases women’s empowerment through knowledge gain, decision-making, and economic stability [27]. A study from Africa indicates that women with more knowledge of family planning can make decisions confidently. [38], which aligns with our study, where participants felt that with complete information about family planning, they could convince their husbands to adopt the specific method. Decision-making power is linked with the knowledge of contraception. The participants in our study also agreed that understanding family planning would increase their self-confidence in the contraceptive method. Evidence also suggests that mobile interventions increase women's decision-making and empowerment [29].

To our knowledge, this is the first exploratory study to assess the feasibility of mHealth interventions using women's perceptions of living in low socio-economic areas of Karachi. There is evidence that mobile phone intervention has been experienced in the field of immunization in Pakistan [30, 31]; however, mHealth for family planning has yet to be experienced in Pakistan. The provider's perspective on the mHealth intervention helped triangulate this study's qualitative data. The study concludes that there is a need for such a mobile application that can fill the knowledge gaps for family planning and improve women's empowerment and decision-making. From the results of this study, it is clear that a mobile application, which has all the information about methods and side effects, will be accepted by women living in low socio-economic areas. Developing a mobile application and testing the application among the population living in low-socioeconomic areas is the way forward for this mHealth intervention.

Initially, the focus group discussions were planned to record participants' views. Due to the COVID situation, these focus groups were converted into in-depth interviews, which is one of the limitations. In the absence of family planning or sexual health application in the local context, it was difficult to explain the application’s content to the women during interviews, another limitation of this study. There is a need for quantitative exploration to quantify the finding of this study. However, participants of this study were women who own smartphones or use their husband's phones, and the perceived benefit of the mHealth intervention was not analyzed separately. Therefore, it is suggested to conduct interventional studies.

## Conclusion

mHealth intervention could answer many challenges faced by the family planning services provided in Pakistan. High penetration of mobile phone among women open venues for cost-effective intervention that can reduce the knowledge gap and address cultural barriers in the country for family planning services. The primary benefit of the mHealth strategy is that it will help to increase women's self-confidence in choosing and deciding about the family planning method. The next step is to implement such an intervention for family planning to identify the impact on the larger population for family planning uptake.

## Data Availability

All the data (used and analyzed) in this study are available from the corresponding author on request.

## Abbreviations

FP: Family planning
iMACC: Mobile application for contraceptive choice
LMIC: Low and middle income countries
LHV: Lady health visitors
LHW: Lady health workers
M4RH: Mobile for reproductive health
